# Supplementary material for: Limited introgression supports division of giraffe into four species
Source: Ecol Evol. 2018 Sep 5;8(20):10156–65. doi: 10.1002/ece3.4490 (PMC6206193; doi:10.1002/ece3.4490)
Supplement: Supplementary file 1 [file ECE3-8-10156-s001.pdf]

Supplemental material

Limited Introgression Supports Division of Giraffe into Four Species

Authors:

Sven Winter[1,2]*, Julian Fennessy[3] & Axel Janke[1,2]

Affiliations:

[1]Senckenberg Biodiversity and Climate Research Centre, Senckenberganlage 25, 60325 Frankfurt am Main, Germany

[2]Goethe University, Institute for Ecology, Evolution & Diversity, Max-von-Laue-Str. 13, 60438 Frankfurt am Main, Germany

[3]Giraffe Conservation Foundation, PO Box 86099, Eros, Windhoek, Namibia, 11005

*corresponding author

sven.winter@senckenberg.de

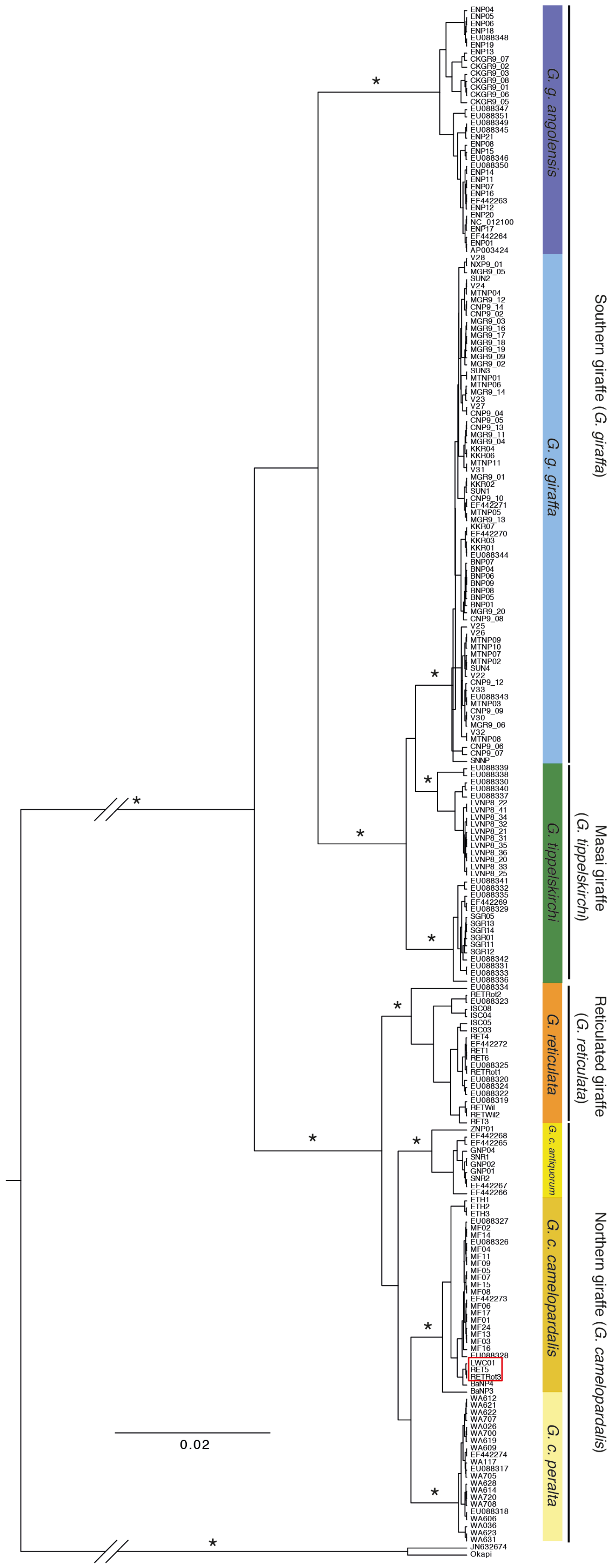

Supplementary Fig. 1. MtDNA Bayesian tree of 217 giraffe individuals

Asterisks show major branches with a posterior probability ≥ 0.95 and the red frame shows potential reticulated giraffe / Nubian giraffe hybrids. Two different okapi individuals were used as an outgroup. Note, the root is not to scale.

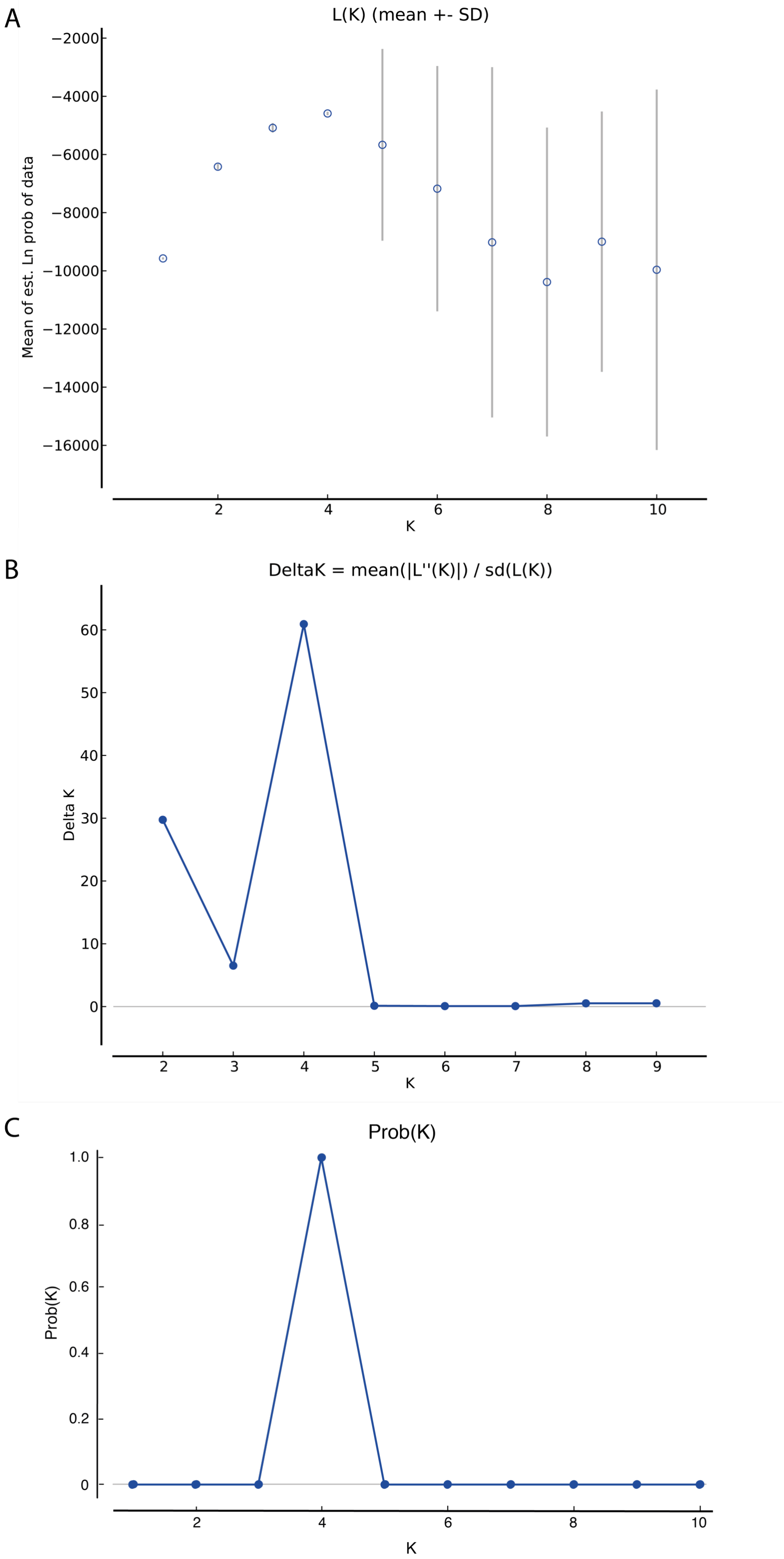

Supplementary Fig. 2. Statistical calculations to evaluate the best fitting K in STRUCTURE

(A) Mean likelihood (L(K)) and variance per K value from STRUCTURE according to (Pritchard, Wen, & Falush, 2010). (B) Delta K plot as per Evanno, Regnaut, & Goudet (2005) to find the best fitting number of populations (K) for the data. K = 4 has the highest Delta K. (C) Probability by K plot according to Pritchard, Stephens, & Donnelly (2000). K = 4 shows the highest probability.

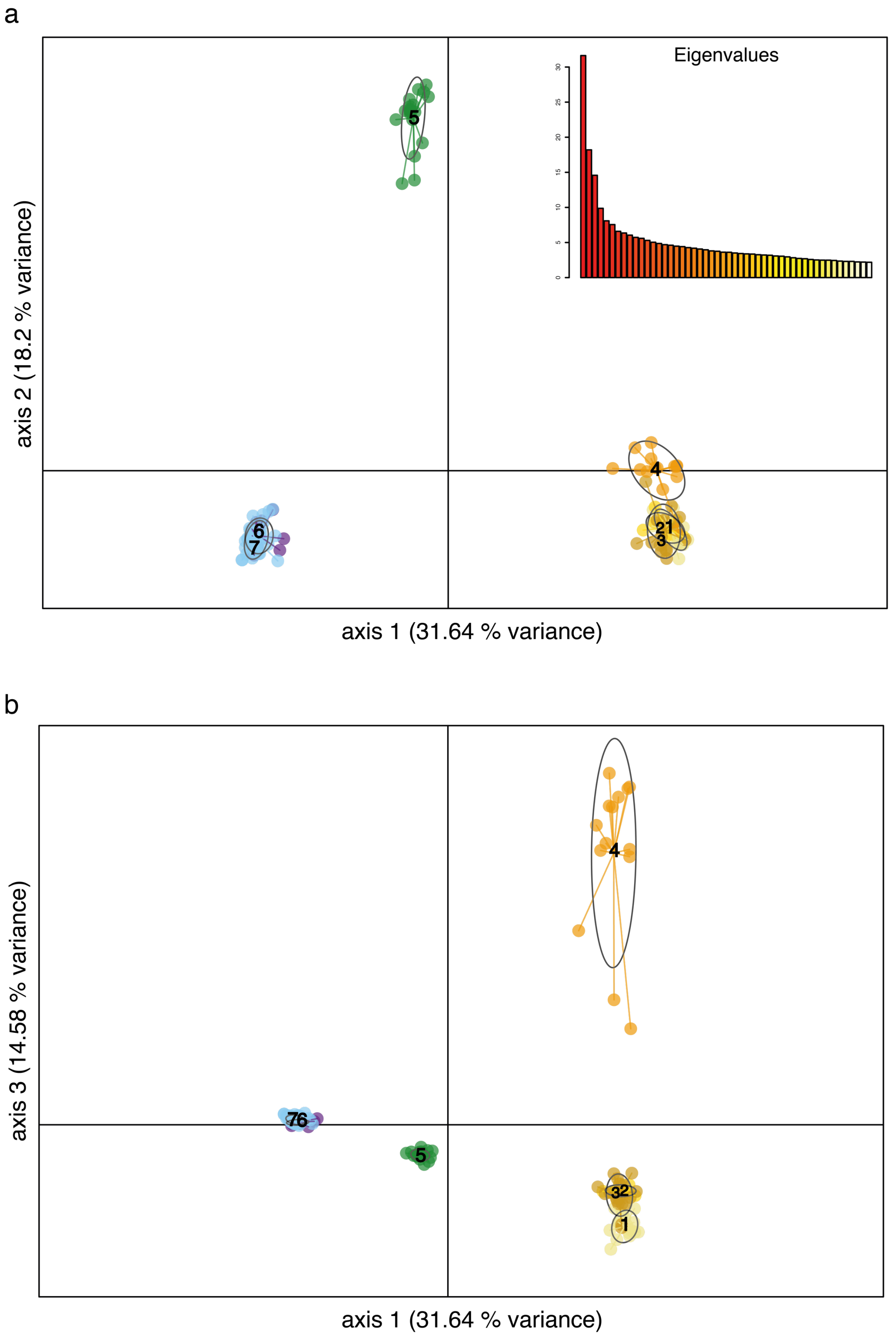

Supplementary Fig. 3. Additional PCAs based on mtDNA clades.
PCA axes 1-2 and axes 1-3 for seven giraffe mtDNA clades (1: West African; 2: Kordofan; 3: Nubian; 4: reticulated; 5: Masai; 6: Angolan; 7: South African). Colored as in Supplementary Fig. 1. The 95% confidential intervals are shown as grey colored oval outlines. Note that the confidence intervals in the PCA axes 1-2, as well as axes 1-3, indicate the same four significantly different clusters as seen in Fig. 3c.

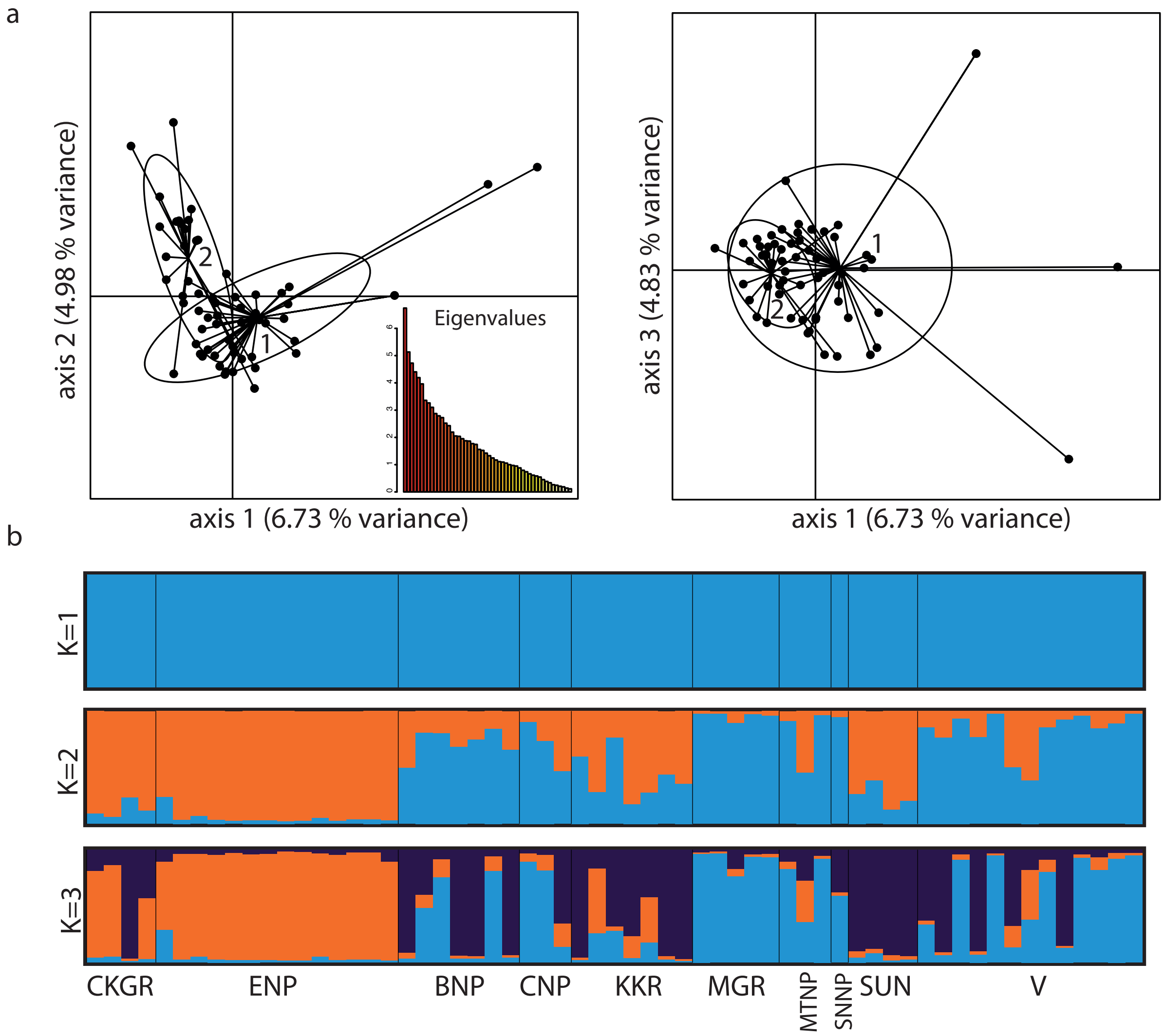

Supplementary Fig. 4. Additional PCAs and STRUCTURE analyses for southern giraffe

(a) PCA axes 1-2 and axes 1-3 for the southern giraffe populations (1: South African giraffe; 2: Angolan giraffe). The 95% confidential intervals are shown as oval outlines. Note, the confidence intervals in the PCA axes 1-2, as well as axes 1-3, indicate no substructure within the southern giraffe.

(b) STRUCTURE analysis for the southern giraffe populations (CKGR and ENP are Angolan giraffe, the remaining populations are South African giraffe) does not show additional clustering but a high level of admixture, which contradicts the clear separation of subspecies by mtDNA. Note, detailed information about the populations are listed in Supplementary Table 1.

a

b

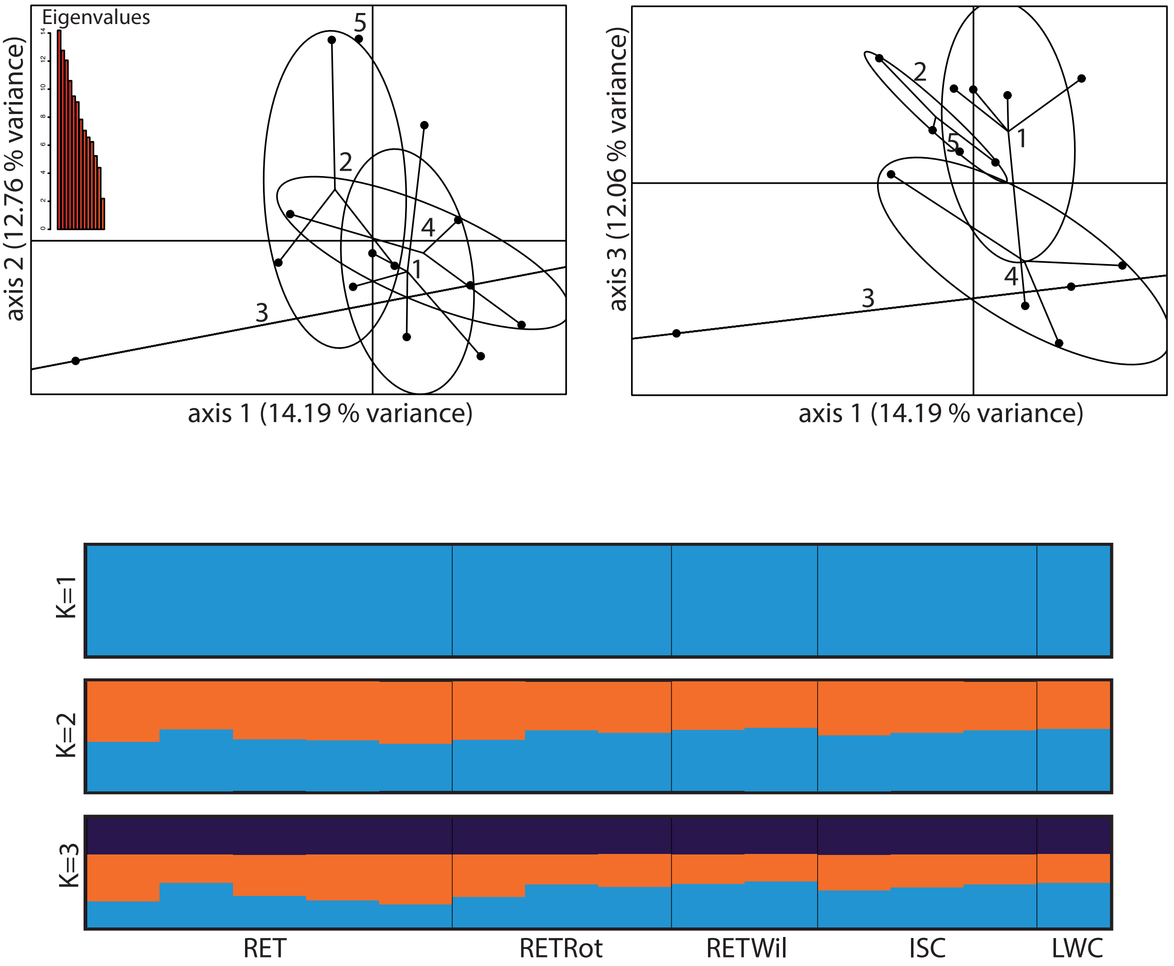

Supplementary Fig. 5. Additional PCAs and STRUCTURE analyses for reticulated giraffe

(a) PCA axes 1-2 and axes 1-3 for the sampled reticulated giraffe populations (1: RET; 2: RETRot; 3: RETWil; 4: ISC; 5: LWC). The 95% confidence intervals are shown as oval outlines. Note that the confidence intervals in the PCA axes 1-2, as well as axes 1-3, indicate no substructure within the reticulated giraffe.

(b) Separate STRUCTURE analysis for reticulated giraffe populations (RET, RETRot and RETWil: captive animals; ISC and LWC: wild populations) shows no additional clustering.

Note, detailed information about the populations are listed in Supplementary Table 1.

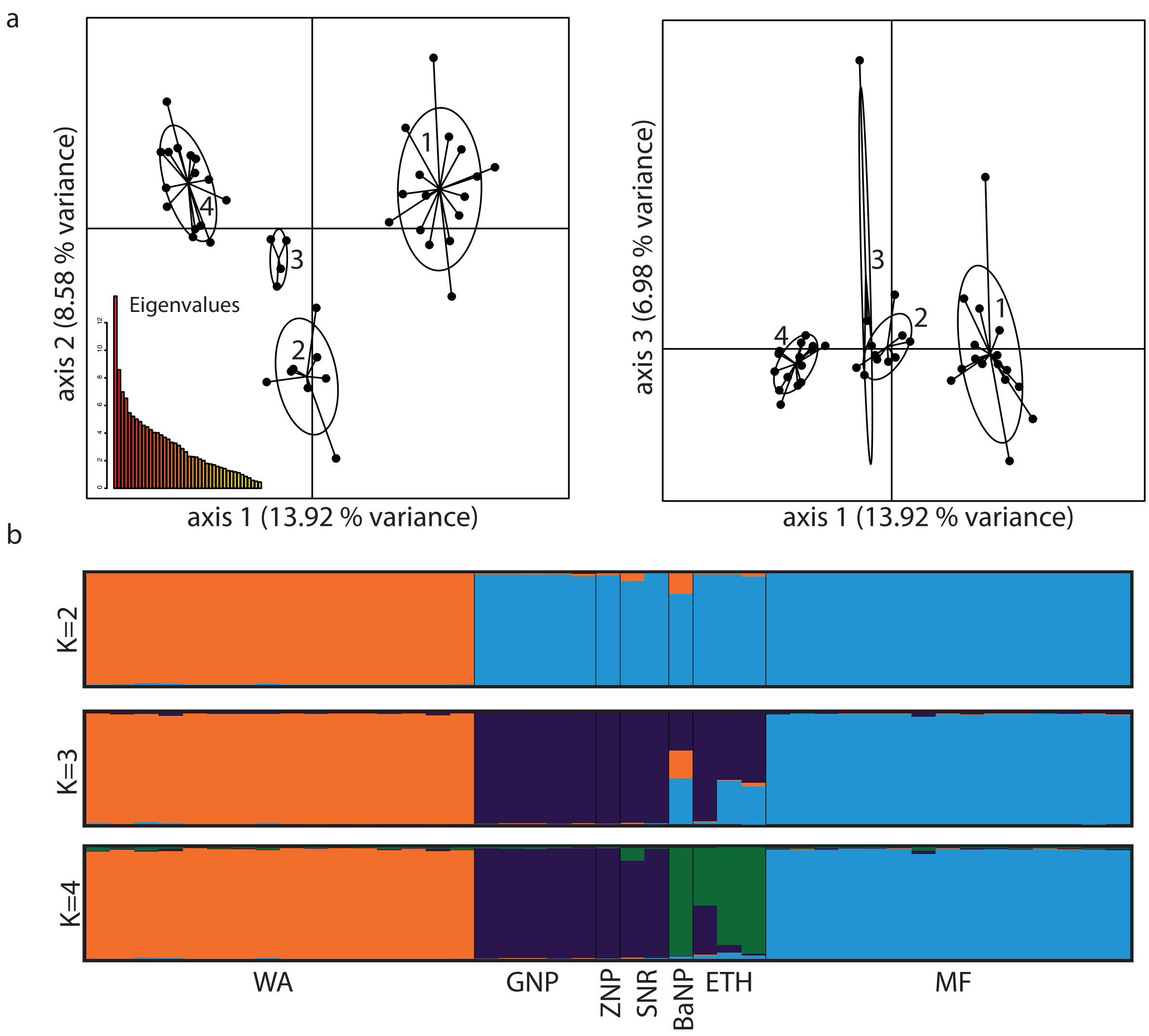

Supplementary Fig. 6. Additional PCAs and STRUCTURE analyses for northern giraffe

(a) PCA axes 1-2 and axes 1-3 for the sampled northern giraffe populations (1: West African giraffe; 2: Kordofan giraffe; 3: Nubian giraffe; 4: former Rothschild's giraffe). The 95% confidence intervals are shown as oval outlines. Note, the confidence intervals in the PCA axes 1-2, as well as axes 1-3, indicate some substructure within the northern giraffe. However, there is no clear distinction between Kordofan and Nubian giraffe.

(b) Separate STRUCTURE analysis for the northern giraffe populations (WA: West African giraffe; GNP, ZNP, and SNR: Kordofan giraffe; BaNP and ETH: Nubian giraffe; MF: former Rothschild's giraffe) shows additional substructure for up to four clusters, but also highlights admixture at K=3.

Note, detailed information about the populations are listed in Supplementary Table 1.

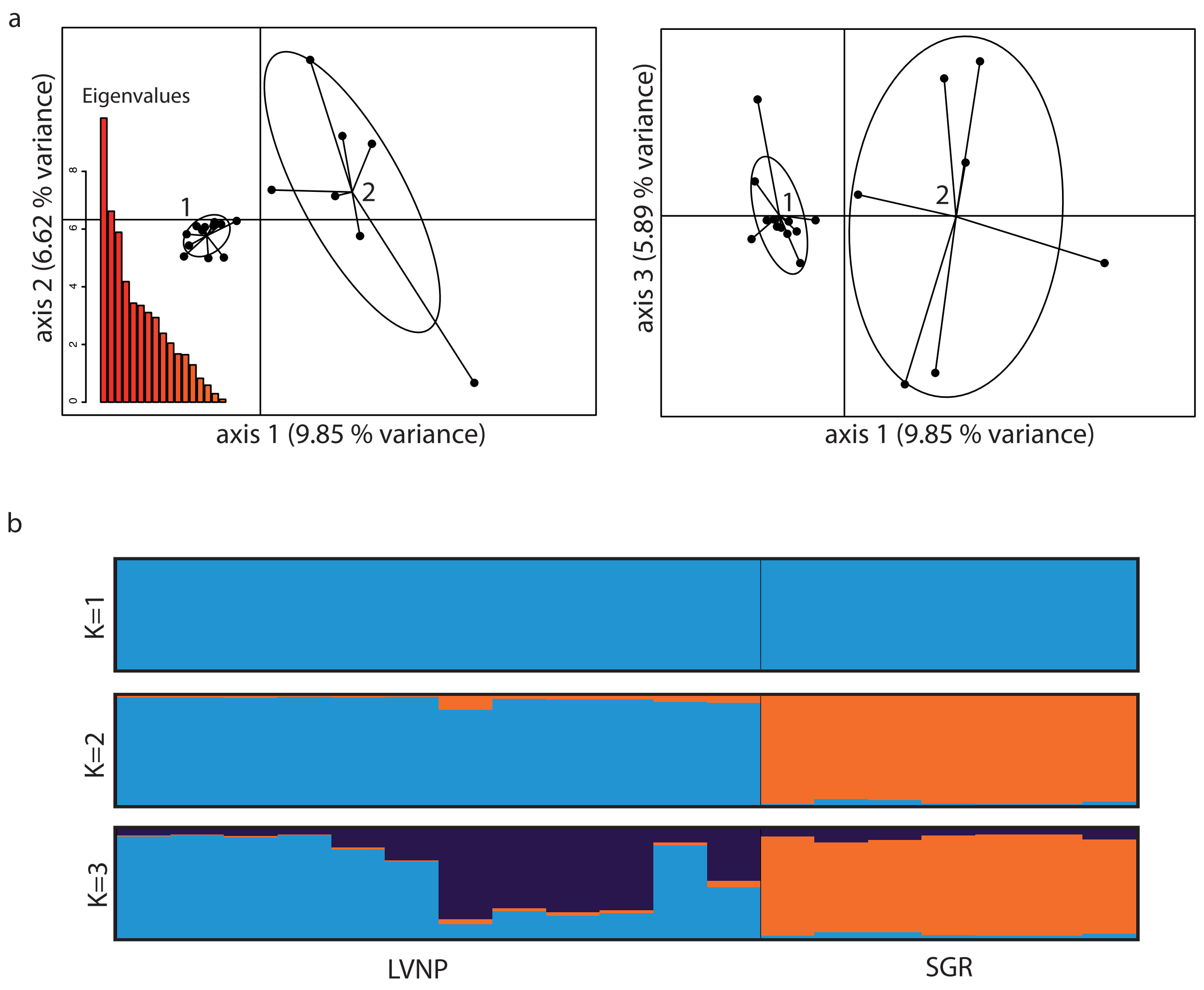

Supplementary Fig. 7. Additional PCAs and STRUCTURE analyses for Masai giraffe

(a) PCA axes 1-2 and axes 1-3 for the two sampled Masai giraffe populations (1: LVNP; 2: SGR). The 95% confidence intervals are shown as oval outlines. Note that the confidence intervals in the PCA axes 1-2, as well as axes 1-3, indicate some substructure within the Masai giraffe.

(b) Separate STRUCTURE analysis for the Masai giraffe populations shows additional clustering for two populations.

Note, detailed information about the populations are listed in Supplementary Table 1.

Supplementary Table 1. Location, abbreviation, number of individuals (n), (sub)species and source of analyzed giraffe and okapi sequences for mtDNA and nuclear analyses.
Individual sample ID´s see Figure 2 and Figure S1. Bold numbers indicate populations newly added in this study and underlined numbers indicate populations with increased sample size.

Location	Abbreviation	n (mtDNA)	n (nuclear DNA)	(Sub)species
Badingilo National Park, South Sudan	BaNP	2	1	*G. c. camelopardalis*
Basel Zoo, Switzerland	Okapi	1	1	*O. johnstoni*
Bwabwata National Park, Namibia	BNP	7	7	*G. g. giraffa*
Central Kalahari Game Reserve, Botswana	CKGR	7	4	*G. g. angolensis*
Chobe National Park, Botswana	CNP	11	3	*G. g. giraffa*
Etosha National Park, Namibia	ENP	17	14	*G. g. angolensis*
Gambella National Park, Ethiopia	ETH	3	3	*G. c. camelopardalis*
Garamba National Park, DR Congo	GNP	5	5	*G. c. antiquorum*
Ishqbini Conservancy, Kenya	ISC	4	**3**	*G. reticulata*
Khamab Kalahari Reserve, South Africa	KKR	6	7	*G. g. giraffa*
Koure, Niger	WA	18	16	*G. c. peralta*
Loisaba Wildlife Conservancy, Kenya	LWC	1	**1**	*G. reticulata*
Luangwa Valley National Park, Zambia	LVNP	11	12	*G. tippelskirchi*
Moremi Game Reserve, Botswana	MGR	16	5	*G. g. giraffa*
Mosi-oa-Tunya National Park, Zambia	MTNP	11	3	*G. g. giraffa*
Murchison Falls National Park, Uganda	MF	16	15	*G. c. camelopardalis*
Nürnberg Zoo, Germany	RET	5	5	*G. reticulata*
Nxai Pans, Botswana	NXP	1	–	*G. g. giraffa*
Rotterdam Zoo, Netherlands	RETRot	**3**	**3**	*G. reticulata*
Selous Game Reserve, Tanzania	SGR	6	7	*G. tippelskirchi*
Shambe National Park, South Sudan	SNR	2	2	*G. c. antiquorum*
Sioma Ngwezi National Park, Zambia	SNNP	1	1	*G. g. giraffa*
Sun hotel, Livingstone, Zambia	SUN	4	4	*G. g. giraffa*
Vumbura Concession, Botswana	V	11	13	*G. g. giraffa*
Wilhelma Stuttgart, Germany	RETWil	2	2	*G. reticulata*
Zakouma National Park, Chad	ZNP	1	1	*G. c. antiquorum*

Additional mtDNA sequences from GenBank

Accession No.	(Sub)species	Source
EF442263 - EF442274	*Giraffa spp.*	Hassanin et al., 2007
EU088317 - EU088320	*Giraffa spp.*	Brown et al., 2007
EU088322 - EU088351	*Giraffa spp.*	Brown et al., 2007
AP003424	*G. g. angolensis*	Yasue et al. unpublished
JN632674	*O. johnstoni*	Hassanin et al., 2012
NC_012100	*G. g. angolensis*	Yasue et al. unpublished

Supplementary Table 2. Giraffe and okapi specific primer sequences and PCR conditions for 14 new nuclear intron loci.

Primer sequences and PCR conditions of the remaining seven intron loci and the mtDNA loci can be found in Bock et al. (2014) and Fennessy et al. (2016).

Name & locus	Primer sequence 5'-3'	PCR conditions
Intron SW05 (RFC5, intron 9, chromosome 17)	for: GATCACTCTGGAACCTGCTCA<br>rev: CATACCTGTGGTTCTGCGGT	TD-PCR (T_a = 70-60 °C; 10 cycles), standard PCR (T_a = 60 °C; 30 cycles)
Intron SW07 (USP33, intron 18, chromosome 3)	for: TGACGACCAGAGTGTCACTG<br>rev: TCTTTTTGTGCTTCTTCACTGCT	TD-PCR (T_a = 67-57 °C; 10 cycles), standard PCR (T_a = 57 °C; 30 cycles)
Intron SW21 (MACF1, intron 46, chromosome 3)	for: GCTCACGACCTCATGGAAAT<br>rev: GTTGGAAATGGCTGAGGATG	TD-PCR (T_a = 66-59 °C; 14 cycles), standard PCR (T_a = 59 °C; 26 cycles)
Intron SW40 (IGF2B1, intron 11, chromosome 19)	for: GGCAGCACATCAAACAGCTC<br>rev: GGGGTCCAGTGATGATGACC	See Intron SW05
Intron SW43 (COL5A2, intron 32, chromosome 2)	for: AATGGCTGGAGGACATGGTC<br>rev: GCCGGAAGTTCCTGCAATTC	See Intron SW05
Intron SW44 (CTAGE5, intron 8, chromosome 21)	for: CCCTCAAATCACAAGTAGCTGA<br>rev: TCTGGCTTTCCTGAAGTTGAGA	See Intron SW05
Intron SW51 (NOTCH2, intron 33, chromosome 3)	for: AAAAATGGGGCCAACCGAGA<br>rev: GGGCAGCAAGAAACAGAGGT	See Intron SW05
Intron SW68 (CCT2, intron 14, chromosome 5)	for: TGAAGGCAAAACAACCGCTG<br>rev: CGCTTCACTTGGAAACTTTCTGT	See Intron SW05
Intron SW84 (DHX36, intron 4, chromosome 1)	for: CATCCTTCCCTGACCTCTCAG<br>rev: TCCACAACCAGTTTCACCACT	See Intron SW05
Intron SW108 (C1orf74, intron 1, chromosome 16)	for: TCCAGTGTTGTTGCTGCTGA<br>rev: TCTGGGAGGACCTCGTTTCT	See Intron SW05
Intron SW111 (SAP130, intron 5, chromosome 2)	for: CCCCCTCTTCACATTGGAGC<br>rev: AACTGGACATCACTGCAGCA	See Intron SW05
Intron SW113 (PLCE1, intron 20, chromosome 26)	for: ACTCTGCTTGCAACAAAGGA<br>rev: TCTGGTGCAATCTGTCTGCT	See Intron SW05
Intron SW117 (DDX1, intron 15, chromosome 11)	for: TTCCTGGTCCTGGATGAAGC<br>rev: AACCTTGAGAAAGAAGCCCAT	See Intron SW05
Intron SW123 (USP54, intron 9, chromosome 28)	for: CCCCCAAGTTGAGTTCCAGT<br>rev: CCGTTGAGGAATCGGTTCGA	See Intron SW05

Note – for: forward primer. rev: reverse primer. TD-PCR: touchdown PCR. T_a: primer annealing temperature. The locus is the gene name of the human orthologs, the respective intron, and the chromosome of *Bos taurus* it is located on.

Supplementary Table 3. List of pairwise F_{st} values for 21 nuclear loci of four giraffe species.

	**Northern**	**Reticulated**	**Masai**	**Southern**
**Northern**	–			
**Reticulated**	0.23725**	–		
**Masai**	0.57289**	0.57813**	–	
**Southern**	0.62072**	0.63408**	0.68790**	–

Note – ** indicates significance of F_{st} values at $p < 0.001$.

Supplementary Table 4. Pairwise F_{st} values for 21 nuclear loci between subspecies / populations within four giraffe species.

a. Pairwise F_{st} values among southern giraffe subspecies

	South African giraffe	Angolan giraffe
**South African giraffe**	–	
**Angolan giraffe**	0.10199**	–

b. Pairwise F_{st} values among reticulated giraffe populations

	RET	ISC	LWC
**RET**	–		
**ISC**	0.04000	–	
**LWC**	0.09065	0.10268	–

c. Pairwise F_{st} values among northern giraffe subspecies (incl. former Rothschild's giraffe)

	West African giraffe	Kordofan giraffe	Nubian giraffe	"Rothschild's giraffe"
**West African giraffe**	–			
**Kordofan giraffe**	0.21326**	–		
**Nubian giraffe**	0.15513**	0.12841**	–	
**"Rothschild's giraffe"**	0.26777**	0.27702**	0.15245**	–

d. Pairwise F_{st} values among Masai giraffe populations

	SGR	LVNP
**SGR**	–	
**LVNP**	0.39517**	–

Note – ** indicates significance of F_{st} values at $p < 0.001$. Detailed information about the populations are listed in Supplementary Table 1.

Supplementary Table 5. Long- and short-term gene flow estimates among four giraffe species.

a. Mutation-scaled effective population size derived from MIGRATE-N of the four giraffe species

Species	Estimated Θ (95 % conf. int.)
Northern giraffe	0.00137 (0.0 – 0.00287)
Reticulated giraffe	0.00110 (0.0 – 0.00267)
Masai giraffe	0.00003 (0.0 – 0.00153)
Southern giraffe	0.00003 (0.0 – 0.00193)

b. Estimates of gene flow derived from MIGRATE-N and BayesAss among the four giraffe species

Migration route	M (95 % conf. int.) [MIGRATE-N]	N_em	m (+/- sdev.) [BayesAss]
Reticulated → Northern	523.3 (253.3 – 793.3)	0.17923	0.0071 (0.0070)
Masai → Northern	190.0 (0.0 – 373.3)	0.06508	0.0071 (0.0069)
Southern → Northern	123.3 (0.0 – 306.7)	0.04223	0.0071 (0.0069)
Northern → Reticulated	3436.7 (3140.0 – 4246.7)	0.94509	0.0209 (0.0196)
Masai → Reticulated	390.0 (120.0 – 693.3)	0.10725	0.0208 (0.0195)
Southern → Reticulated	376.7 (0.0 – 660.0)	0.10359	0.0208 (0.0196)
Northern → Masai	276.7 (20.0 – 553.3)	0.00208	0.0144 (0.0138)
Reticulated → Masai	796.7 (366.7 – 1313.3)	0.00598	0.0145 (0.0139)
Southern → Masai	503.3 (186.7 – 786.7)	0.00377	0.0144 (0.0138)
Northern → Southern	176.7 (0.0 – 360.0)	0.00133	0.0052 (0.0051)
Reticulated → Southern	176.7 (0.0 – 366.7)	0.00133	0.0051 (0.0051)
Masai → Southern	243.3 (6.7 – 493.3)	0.00182	0.0051 (0.0050)

Supplementary Table 6. Long-term and short-term gene flow estimates between giraffe subspecies (populations) within species.

a. Mutation-scaled effective population size derived from MIGRATE-N for subspecies (populations) within giraffe species

Species	Estimated Θ (95 % conf. int.)
West African giraffe	0.00130 (0.0 – 0.00287)
Kordofan giraffe	0.00150 (0.0 – 0.00307)
Nubian giraffe	0.00110 (0.0 – 0.00273)
South African	0.00137 (0.0 – 0.00313)
Angolan	0.00050 (0.0 – 0.00213)
SGR	0.00143 (0.0 – 0.00300)
LVNP	0.00097 (0.0 – 0.00260)

b. Estimates of gene flow derived from MIGRATE-N and BayesAss among subspecies (populations) within species

Migration route	M (95 % conf. int.) [MIGRATE-N]	N_em	m (+/- sdev.) [BayesAss]
Kordofan → West African	1611.7 (873.3 – 2563.3)	0.52380	0.0211 (0.0168)
Nubian → West African	2631.7 (1756.7 – 3520.0)	0.85530	0.0150 (0.0141)
West African → Kordofan	3285.0 (2413.3 – 4186.7)	1.23188	0.0636 (0.0364)
Nubian → Kordofan	4215.0 (3523.3 – 4990.0)	1.58063	0.0386 (0.0344)
West African → Nubian	2301.7 (1516.7 – 3193.3)	0.63298	0.0168 (0.0158)
Kordofan → Nubian	1438.3 (863.3 – 2276.7)	0.39553	0.0347 (0.0228)
South African → Angolan	8130.0 (6773.3 – 9420.0)	1.01625	0.0521 (0.0328)
Angolan → South African	8456.7 (7913.3 – 9446.7)	2.89642	0.0223 (0.0154)
Masai			
SGR → LVNP	1610.0 (940.0 – 2793.3)	0.39043	0.0239 (0.0221)
LVNP → SGR	8250.0 (6853.3 – 9746.7)	2.94938	0.0405 (0.0362)

Supplemental references

Bock, F., Fennessy, J., Bidon, T., Tutchings, A., Marais, A., Deacon, F., Janke, A., 2014. Mitochondrial sequences reveal a clear separation between Angolan and South African giraffe along a cryptic rift valley. BMC Evol. Biol. 14, 1–12. https://doi.org/10.1186/s12862-014-0219-7

Brown, D.M., Brenneman, R.A., Koepfli, K.-P., Pollinger, J.P., Milá, B., Georgiadis, N.J., Louis, E.E., Grether, G.F., Jacobs, D.K., Wayne, R.K., 2007. Extensive population genetic structure in the giraffe. BMC Biol. 5, 1–13. https://doi.org/10.1186/1741-7007-5-57

Evanno, G., Regnaut, S., Goudet, J., 2005. Detecting the number of clusters of individuals using the software structure: a simulation study. Mol. Ecol. 14, 2611–2620. https://doi.org/10.1111/j.1365-294X.2005.02553.x

Fennessy, J., Bidon, T., Reuss, F., Kumar, V., Elkan, P., Nilsson, M.A., Vamberger, M., Fritz, U., Janke, A., 2016. Multi-locus Analyses Reveal Four Giraffe Species Instead of One. Curr. Biol. 26, 2543–2549. https://doi.org/10.1016/j.cub.2016.07.036

Hassanin, A., Delsuc, F., Ropiquet, A., Hammer, C., Jansen van Vuuren, B., Matthee, C., Ruiz-Garcia, M., Catzeflis, F., Areskoug, V., Nguyen, T.T., Couloux, A., 2012. Pattern and timing of diversification of Cetartiodactyla (Mammalia, Laurasiatheria), as revealed by a comprehensive analysis of mitochondrial genomes. C. R. Biol. 335, 32–50. https://doi.org/10.1016/j.crvi.2011.11.002

Hassanin, A., Ropiquet, A., Gourmand, A.-L., Chardonnet, B., Rigoulet, J., 2007. Mitochondrial DNA variability in Giraffa camelopardalis: consequences for taxonomy, phylogeography and conservation of giraffes in West and central Africa. C. R. Biol. 330, 265–274. https://doi.org/10.1016/j.crvi.2007.02.008

Pritchard, J., Wen, X., Falush, D., 2010. Documentation for STRUCTURE software, version 2.3. University of Chicago, Chicago, IL.

Pritchard, J.K., Stephens, M., Donnelly, P., 2000. Inference of Population Structure Using Multilocus Genotype Data. Genetics 155, 945–959.
